# Correlates of infection with *Helicobacter pylori* positive and negative cytotoxin-associated gene A phenotypes among Arab and Jewish residents of Jerusalem

**DOI:** 10.1017/S0950268819001456

**Published:** 2019-09-25

**Authors:** K. Muhsen, R. Sinnereich, G. Beer-Davidson, H. Nassar, W. Abu Ahmed, D. Cohen, J. D. Kark

**Affiliations:** 1 Department of Epidemiology and Preventive Medicine, School of Public Health, Sackler Faculty of Medicine, Tel Aviv University Ramat Aviv, Tel Aviv, Israel; 2 Hebrew University-Hadassah School of Public Health and Community Medicine, Jerusalem, Israel; 3 St. Joseph Hospital, East Jerusalem and Department of Cardiology, Hadassah-Hebrew University Medical Center, Ein Kerem, Jerusalem 91120, Israel

**Keywords:** Epidemiology, *Helicobacter pylori*, public health, serology, cytotoxin-associated gene A (CagA)

## Abstract

We examined the prevalence and correlates of *Helicobacter pylori* (*H. pylori*) infection according to cytotoxin-associated gene A (CagA) phenotype, a main virulence antigen, among the ethnically diverse population groups of Jerusalem. A cross-sectional study was undertaken in Arab (*N* = 959) and Jewish (*N* = 692) adults, randomly selected from Israel's national population registry in age-sex and population strata. Sera were tested for *H. pylori* immunoglobulin G (IgG) antibodies. Positive samples were tested for virulence IgG antibodies to recombinant CagA protein, by enzyme-linked immunosorbent assay. Multinomial regression models were fitted to examine associations of sociodemographic factors with *H. pylori* phenotypes. *H. pylori* IgG antibody sero-prevalence was 83.3% (95% confidence interval (CI) 80.0%–85.5%) and 61.4% (95% CI 57.7%–65.0%) among Arabs and Jews, respectively. Among *H. pylori* positives, the respective CagA IgG antibody sero-positivity was 42.3% (95% CI 38.9%–45.8%) and 32.5% (95% CI 28.2%–37.1%). Among Jews, being born in the Former Soviet Union, the Middle East and North Africa, *vs.* Israel and the Americas, was positively associated with CagA sero-positivity. In both populations, sibship size was positively associated with both CagA positive and negative phenotypes; and education was inversely associated. In conclusion, CagA positive and negative infection had similar correlates, suggesting shared sources of these two *H. pylori* phenotypes.

## Introduction

The bacterium *Helicobacter pylori* (*H. pylori*) colonises the stomach and causes persistent infection [1]. *H. pylori* infection is highly prevalent in developing countries, reaching 90% in adults, as compared with 20%–50% in developed countries [1]. In the latter, the prevalence of *H. pylori* infection is high among immigrants from endemic regions, and in ethnic groups of low socioeconomic status [2–6]. The risk factors for *H. pylori* infection involve living conditions in childhood, a sensitive period of acquiring the infection [7]; and include low parental education, crowded households and large families [2, 6–8]. *H. pylori*-infected parents and siblings comprise a main reservoir of the infection, and they increase the likelihood of its acquisition in young children [4, 9].

In a subset of *H. pylori*-infected persons, *H. pylori* causes gastric and duodenal ulcers, gastric mucosa-associated lymphoid tissue lymphoma and gastric cancer [1, 10]. *H. pylori* virulence factors, host-related factors, environmental characteristics and the interactions among them are involved in the development of *H. pylori*-gastroduodenal illness (reviewed in [11]). Cytotoxin-associated gene A (CagA) antigen has been studied widely as a virulence factor of *H. pylori*. The majority of *H. pylori* strains carry the *cag* pathogenicity island that encodes for a type-IV secretory apparatus through which CagA protein is inserted into the host cell (reviewed by Surbaum and Michetti [1]). Infection with *H. pylori* CagA positive strains is associated with increased risk for peptic ulcer disease, premalignant gastric lesions and gastric cancer [10, 12]. While other *H. pylori* antigens have recently studied, only a few showed positive associations with gastric cancer [13–15], and the association between CagA sero-positivity and gastric cancer was of greater magnitude than other antigens [13, 14]. Moreover, following adjustment for the presence of other antigens, CagA remained the only antigen associated with an increased risk of gastric cancer [14]. Therefore, understanding the correlates of *H. pylori* infection according to CagA phenotype is important for prevention of gastric cancer. It is not clear whether there are specific risk factors for *H. pylori* phenotypes, i.e. for CagA positive strains in contrast to CagA negative strains. Moreover, in recent decades, the prevalence of *H. pylori* infection has declined [16], as has the burden of its associated diseases (i.e. peptic ulcer and gastric cancer) in developed countries [17, 18]; the prevalence of CagA positive strains has declined more rapidly than CagA negative strains [19]. In parallel, populations have migrated from low-middle income countries, usually with a high prevalence of *H. pylori* infection, to high-income countries. These changes require reassessment of risk factors for *H. pylori* infection, especially infections with CagA virulent strains.

Israel, which is presently a high-income country with a secure water supply, provides a unique setting to address the abovementioned topics, due to the multiethnic composition of the population, which consists of Jews and Arabs. The Jewish population in Israel is heterogeneous in terms of country of birth, due to large waves of immigration from around the world over the past seven decades. In this study, we examined the prevalence and sociodemographic correlates of *H. pylori* infection, specifically by CagA phenotype, among Jewish and Arab adults living in Jerusalem.

## Materials and methods

### Study design and population

A sero-epidemiological study was performed using stored anonymised (coded) serum samples obtained in the framework of a cross-sectional study among Jewish and Arab residents of Jerusalem, aged 25–78 years at examination [20, 21]. Details on the original study design have been reported [20, 21]. Briefly, age-sex-stratified random samples comprising 2000 Arab residents of East Jerusalem and 2000 Israeli Jews living in Jerusalem were drawn from the Israel national population registry. The population registry of Israel contains information on all residents including their ethnicity, sex and date of birth. Persons were ineligible to participate if they were unable to provide informed consent, institutionalised, housebound or had a severe illness. Additionally, women were ineligible if they were pregnant or gave birth within 3 months prior to study enrolment. The response rates among those recruited were 77% and 54% for Arabs (*N* = 970) and Jews (*N* = 712), respectively [20, 21].

A standardised protocol and questionnaires were applied in data and biological sample collection [20, 21]. Information was collected on age (grouped as 25–34, 35–44, 45–54, 55–64 and 65–78 years); sex, marital status (married or cohabiting and unmarried (single, widowed or separated)); number of siblings (grouped as 0–3, 4–7, ⩾8), religiosity; and education (grouped as having an academic degree/education, high-school/some college, some high school or less). For Jewish participants, country of birth was defined based on the participant's report on his/her country of birth, which was classified as Israel, Former Soviet Union (FSU), Eastern Europe, rest of Europe, Asia (mainly the Middle East), North Africa and the Americas. Of the Arab participants, 97.4% were born in Israel, 1.8% in Jordan and 0.8% in other countries. Therefore, the analysis according to country of birth was not applicable to Arab participants. The rationale behind focusing on these sociodemographic factors is that they were shown to be associated with *H. pylori* infection in general [2, 3, 5], and with CagA sero-positivity [22], specifically; hence, we aimed to assess whether such correlates differ according to *H. pylori* CagA phenotype. Since *H. pylori* infection is acquired in early childhood [7], adulthood lifestyle characteristics likely do not affect the risk of acquisition of the infection.

### Laboratory methods

Sera stored at −70 °C were thawed and tested for the presence of specific *H. pylori* immunoglobulin G (IgG) antibodies using an enzyme-linked immunosorbent assay (ELISA) (Enzygnost® Anti-*Helicobacter pylori* II/IgG kit, Siemens Diagnostics Product GmbH, Marburg, Germany). The sensitivity and specificity values of the kit are in the range of 94%–98% (according to the manufacturer's instructions). The presence of IgG antibodies against recombinant CagA protein [10], kindly provided by GI Perez-Perez and MJ Blaser (NYU), was measured in *H. pylori-*positive sera using a modified in-house ELISA protocol [10]. Corrected optical density (OD) was calculated as OD at 405 nm minus OD at 620 nm (background). The mean corrected OD (0.07) plus three standard deviations [s.d.] (1 s.d. = 0.06) of 42 serum samples of persons known to be *H. pylori* negative was employed as the cutoff to determine CagA IgG sero-positivity; this cutoff value was 0.254. Results within 0.03 OD units from 0.254 were considered equivocal. Therefore, ODs of 0.285 or greater, in the range of 0.224–0.284 and lower than 0.224 were considered as positive, equivocal and negative for CagA IgG antibodies, respectively. These cutoffs correctly identified all positive and negative controls.

Participants were classified as: (a) *H. pylori* seronegative; (b) *H. pylori* positive, CagA negative, if they had *H. pylori* IgG antibodies, but lacked CagA IgG antibodies or (c) *H. pylori* positive CagA positive if they were positive for *H. pylori* and for CagA IgG antibodies.

### Statistical analysis

Chi-square tests were used to examine unadjusted associations of sociodemographic variables with *H. pylori* IgG-sero-positivity and CagA IgG antibody sero-positivity. Effect modification by population group was assessed using the chi-square test for heterogeneity. Multivariable analyses using multinomial logistic regression models were fitted. In these models, we examined associations of sociodemographic variables with *H. pylori* plus CagA IgG antibody sero-positivity, and with *H. pylori* IgG antibody sero-positivity in the absence of CagA IgG antibodies; the reference group was *H. pylori* sero-negativity. The main independent variable was population group, and the remaining variables were considered as covariates, regardless of statistical significance. Adjusted odds ratios (ORs) and 95% confidence intervals (CIs) were obtained from these models. We conducted both stratified analyses by population group (Jews or Arabs) and pooled analyses. In pooled analyses, interactions between population group and the other sociodemographic characteristics were assessed. Nominal *P* values <0.05 were considered statistically significant. Data were analysed using SPSS version 23 (Armonk, New York, USA) and Winpepi [23].

### Ethics statement

The study was approved by the Institutional Review Board of the Hadassah Medical Center, Jerusalem, and by the ethics committee at Tel Aviv University.

## Results

Sera from 692 Jewish and 959 Arab participants were available for *H. pylori* IgG testing. The mean ages of the Jewish and Arab participants were 52.5 years (s.d. 13.5) and 52.0 years (s.d. 13.9), respectively, *P* = 0.4. Jews and Arabs were also similar with respect to the sex distribution (52.7% and 53.1%, respectively, were males, *P* = 0.8). A lower percentage of the Arabs than Jews had an academic degree: 13.5% *vs.* 37.2%, *P* < 0.001.

Among the Jewish participants, 61.4% (95% CI 57.7%–65.0%) were *H. pylori* positive compared with 83.3% (95% CI 80.0%–85.5%) among the Arabs, *P* < 0.001. Associations of the covariates examined with total *H. pylori* IgG sero-positivity by population group are presented in Supplementary Table S1.

### Correlates of *H. pylori* infection by CagA IgG antibody sero-positivity

Overall, among *H. pylori* sero-positives, 476 (38.9%) tested positive for CagA IgG antibodies and 54 (4.4%) had equivocal results. Among *H. pylori* positive Jewish participants, 138 (32.5% ((95% CI 28.2%–37.1%)) and 15 (3.5%) had positive and equivocal results for CagA IgG antibody, respectively, compared with 338 (42.3% (95% CI 38.9%–45.8%)) and 39 (4.9%), respectively, among *H. pylori* positive Arab residents (*P* < 0.001 for the difference in virulence between the population groups). In further analyses, equivocal results were classified as CagA IgG sero-negatives.

In a population-pooled bivariate analysis, married persons had a slightly higher prevalence of *H. pylori* infection, both CagA IgG sero-positive and CagA negative, compared with unmarried persons. Prevalences of *H. pylori* infection of both phenotypes were inversely related to education and positively related to the number of siblings. No statistically significant difference was observed in *H. pylori* sero-status according to sex, age or religiosity (Table 1).

**Table 1.** Sero-prevalence of *H. pylori* immunoglobulin G antibodies according to CagA status and sociodemographic characteristics^a^

	Total (*n* = 1651)			
	*H. pylori* positive CagA positive	*H. pylori* positive CagA negative	*H. pylori* sero-negative	*P*
Total	476 (28.8%)	748 (45.3%)	427 (25.9%)	
Population				
Jews	138 (19.9%)	287 (41.5%)	267 (38.6%)	<0.001
Arabs	338 (35.2%)	461 (48.1%)	160 (16.7%)	
Sex				
Men	248 (28.4%)	401 (45.9%)	225 (25.7%)	0.8
Women	228 (29.3%)	347 (44.7%)	202 (26.0%)	
Age (years)^b^				
25–34	68 (31.2%)	93 (42.7%)	57 (26.1%)	0.9
35–44	90 (27.6%)	145 (44.5%)	91 (27.9%)	
45–54	107 (30.1%)	165 (46.3%)	84 (23.6%)	
55–64	109 (29.1%)	170 (45.3%)	96 (25.6%)	
65–78	102 (27.1%)	175 (46.5%)	99 (26.3%)	
Marital status^c^				
Married	380 (29.4%)	599 (46.4%)	312 (24.2%)	0.013
Unmarried	95 (26.8%)	146 (41.2%)	113 (31.9%)	
Education^d^				
Some high school or less	293 (33.3%)	423 (48.1%)	163 (18.5%)	<0.001
High school certificate/some college	109 (28.7%)	170 (44.7%)	101 (26.6%)	
Academic education	73 (19.0%)	153 (39.7%)	159 (41.3%)	
Religiosity				
Religious/very religious	187 (30.1%)	265 (42.6%)	170 (27.3%)	0.19
Traditional/secular	285 (28.0%)	480 (47.2%)	253 (24.9%)	
Number of siblings^d^				
0–3	97 (20.4%)	163 (34.3%)	215 (45.3%)	<0.001
4–7	181 (28.4%)	326 (51.1%)	131 (20.5%)	
⩾8	196 (36.9%)	258 (48.6%)	77 (14.5%)	

a *P* value was obtained by the chi-square test.

b *P* for trend = 0.26.

c Married includes also persons who classified themselves as having a partner. The unmarried group includes persons who reported that they are divorced, widowed or separated.

d *P* for trend <0.001.

Information was missing on marital status, education, religiosity and number of siblings for 6 (0.4%), 7 (0.4%), 11 (0.7%) and 7 (0.4%), participants, respectively.

A population-stratified analysis showed that among Jewish participants, associations of education and sibship size with both *H. pylori* phenotypes examined remained statistically significant. Significant (*P* < 0.001) differences in CagA sero-positivity were evident according to country of birth, with the highest prevalence found among the Asian-born (33.3%), followed by those from the FSU (28.9%), North-African, Eastern Europe-born, Israeli-born and born in other regions of Europe; and the lowest observed among those born in the Americas (4.5%) (Table 2).

**Table 2.** Population-stratified analysis of the sero-prevalence of *H. pylori* immunoglobulin G antibodies according to CagA status and sociodemographic characteristics^a^

	Jews (*n* = 692)				Arabs (*n* = 959)				
	*H. pylori* positive CagA positive	*H. pylori* positive CagA negative	*H. pylori* sero-negative	*P*	*H. pylori* positive CagA positive	*H. pylori* positive CagA negative	*H. pylori* sero-negative	*P*	*P* for interaction
Total	138 (19.9%)	287 (41.5%)	267 (38.6%)		338 (35.2%)	461 (48.1%)	160 (16.7%)		
Sex									
Men	79 (21.6%)	150 (41.1%)	136 (37.3%)	0.4	169 (33.2%)	251 (49.3%)	89 (17.5%)	0.3	0.19
Women	59 (18.0%)	137 (41.9%)	131 (40.1%)		169 (37.6%)	210 (46.7%)	71 (15.8%)		
Age (years)^b^									
25–34	16 (18.2%)	32 (36.4%)	40 (45.5%)	0.4	52 (40.0%)	61 (46.9%)	17 (13.1%)	0.8	0.4
35–44	24 (18.6%)	47 (36.4%)	58 (45.0%)		66 (33.5%)	98 (49.7%)	33 (16.8%)		
45–54	34 (23.1%)	63 (42.9%)	50 (34.0%)		73 (34.9%)	102 (48.8%)	34 (16.3%)		
55–64	35 (20.2%)	79 (45.7%)	59 (34.1%)		74 (36.6%)	91 (45.0%)	37 (18.3%)		
65–78	29 (18.7%)	66 (42.6%)	60 (38.7%)		73 (33.0%)	109 (49.3%)	39 (17.6%)		
Marital status^c^									
Married	107 (21.1%)	210 (41.3%)	191 (37.6%)	0.4	273 (34.9%)	389 (49.7%)	121 (15.5%)	0.031	0.13
Unmarried	31 (17.1%)	76 (42.0%)	74 (40.9%)		64 (37.0%)	70 (40.5%)	39 (22.5%)		
Education^d^									
Some high school or less	59 (22.3%)	134 (50.8%)	71 (26.9%)	<0.001	234 (38.0%)	289 (47.0%)	92 (15.0%)	0.002	0.078
High school certificate/some college	37 (22.0%)	62 (36.9%)	69 (41.1%)		72 (34.0%)	108 (50.9%)	32 (15.1%)		
Academic education	42 (16.4%)	90 (35.2%)	124 (48.4%)		31 (24.0%)	63 (48.8%)	35 (27.1%)		
Religiosity									
Religious/ very religious	49 (18.7%)	101 (38.5%)	112 (42.7%)	0.19	138 (38.3%)	164 (45.6%)	58 (16.1%)	0.2	0.2
Traditional/secular	87 (20.5%)	185 (43.6%)	152 (35.8%)		198 (33.3%)	295 (49.7%)	101 (17.0%)		
Number of siblings^e^									
0–3	68 (17.7%)	126 (32.8%)	190 (45.9%)	<0.001	29 (31.9%)	37 (40.7%)	25 (27.5%)	0.006	0.6
4–7	42 (20.3%)	111 (53.6%)	54 (26.1%)		139 (32.3%)	215 (49.9%)	77 (17.9%)		
⩾8	27 (28.1%)	49 (51.0%)	20 (20.8%)		169 (38.9%)	209 (48.0%)	57 (13.1%)		
Country of birth ^f^									
Israel	61 (16.9%)	155 (43.1%)	144 (40.0%)	<0.001	–	–	–		
FSU	24 (28.9%)	35 (42.2%)	24 (28.9%)		–	–	–		
East Europe	7 (22.6%)	11 (35.5%)	13 (41.9%)		–	–	–		
Rest of Europe	7 (16.3%)	14 (32.6%)	22 (51.2%)		–	–	–		
Asia	21 (33.3%)	29 (46.0%)	13 (20.6%)		–	–	–		
Africa	16 (24.2%)	35 (53.0%)	15 (22.7%)		–	–	–		
Americas	2 (4.5%)	8 (18.2%)	34 (77.3%)		–	–	–		

a *P* value was obtained by the chi-square test, unless specified otherwise.

b *P* for trend = 0.26 for Jews and Arabs.

c Married includes also persons who classified themselves as having a partner. The unmarried group includes persons who reported that they are divorced, widowed or separated.

d *P* for trend <0.001 and 0.002, for Jews and Arabs, respectively. *P* for the interaction between population group and each independent variable, by multinomial logistic regression model.

e *P* for trend <0.001 for Jews and Arabs.

f FSU, Former Soviet Union.

Among Jewish participants, information was missing on marital status, education, religiosity, number of siblings and country of birth for 3 (0.4%), 4 (0.6%), 6 (0.9%), 5 (0.7%) and 2 (0.3%), respectively. Among Arab participants, information was missing on marital status, education, religiosity and number of siblings for 3 (0.3%), 3 (0.3%), 5 (0.5%) and 2 (0.2%), respectively.

Among Arabs, the prevalence of CagA IgG serum antibody decreased as educational level increased, and the positive associations of sibship size with both *H. pylori* phenotypes was significant. The association between *H. pylori* infection and marital status was significant only among Arab participants (Table 2) (*P* = 0.13 by chi-square for heterogeneity according to population group).

### Multivariable analysis

In a pooled multivariable multinomial logistic regression model, the associations of population group (Arab *vs.* Jewish participants) with both *H. pylori* phenotypes examined were attenuated after adjustment for education, sibship size and additional covariates, but remained significant (Table 3). Compared with Jewish participants, Arab participants had a twofold higher likelihood to be CagA IgG antibody sero-positive and a 1.5-fold higher likelihood to be positive for *H. pylori* IgG antibody and lacking CagA IgG antibody. The associations of number of siblings and education with *H. pylori* infection (both phenotypes) were also attenuated in multivariable analysis, but remained significant (Table 3). No significant interactions were found between population group with age (*P* = 0.6), number of siblings (*P* = 0.8) or education (*P* = 0.3). An interaction between population group and marital status indicated that married Arab participants had higher odds for infection with CagA negative strains (adjusted OR 1.95 (95% CI 1.07–3.55, *P* = 0.029)).

**Table 3.** Associations of sociodemographic characteristics with *H. pylori* phenotypes (CagA positive and CagA negative) compared with *H. pylori* sero-negatives-pooled multinomial logistic regression model

	*H. pylori* positive CagA positive	*H. pylori* positive CagA negative	*H. pylori* positive CagA positive		*H. pylori* positive CagA negative	
	Unadjusted OR (95% CI)	Unadjusted OR (95% CI)	Adjusted OR (95% CI)	*P*	Adjusted OR (95% CI)	*P*
Population	Overall *P* < 0.001, DF = 2		Overall *P* < 0.001, DF = 2			
Jews	1	1	1		1	
Arabs	4.09 (3.09–5.04)	2.68 (2.10–3.42)	2.31 (1.66–3.21)	<0.001	1.47 (1.10–1.97)	0.01
Age (years)	Overall *P* = 0.9 DF = 8		Overall *P* = 0.6, DF = 8			
25–34	1.16 (0.74–1.81)	0.92 (0.61–1.39)	1.16 (0.72–1.88)	0.5	0.90 (0.58–1.40)	0.6
35–44	0.96 (0.64–1.43)	0.90 (0.63–1.29)	0.80 (0.52–1.24)	0.3	0.72 (0.49–1.06)	0.098
45–54	1.24 (0.83–1.84)	1.11 (0.78–1.59)	1.15 (0.75–1.77)	0.5	0.97 (0.66–1.43)	0.8
55–64	1.10 (0.75–1.63)	1.00 (0.71–1.42)	1.11 (0.73–1.70)	0.6	0.94 (0.65–1.38)	0.7
65–78	1	1	1		1	
Marital status^a^	Overall *P* = 0.015, DF = 2		Overall *P* = 0.2, DF = 2			
Unmarried	1	1	1	0.2	1	0.072
Married	1.45 (1.06–1.98)	1.48 (1.12–1.97)	1.20 (0.86–1.68)		1.32 (0.98–1.78)	
Education	Overall *P* < 0.001, DF = 4		Overall *P* = 0.004, DF = 4			
Some high school or less	3.92 (2.80–5.48)	2.70 (2.03–3.59)	2.11 (1.45–3.07)	<0.001	1.52 (1.10–2.10)	0.011
High school certificate/some college	2.35 (1.60 (3.46)	1.75 (1.26–2.44)	1.71 (1.13–2.57)	0.011	1.31 (0.92–1.86)	0.13
Academic education	1	1	1		1	
Number of siblings	Overall *P* < 0.001, DF = 4		Overall *P* < 0.001, DF = 4			
0–3	1	1	1		1	
4–7	3.06 (2.20–4.25)	3.28 (2.46–4.38)	1.70 (1.16–2.47)	0.005	2.42 (1.75–3.36)	<0.001
⩾8	5.64 (3.95–8.06)	4.42 (3.19–6.12)	2.63 (1.70–4.05)	<0.001	3.00 (2.02–4.45)	<0.001

OR: odds ratio; CI, confidence interval; CagA, cytotoxin-associated gene A; DF, degrees of freedom. Model adjusted for the variables in the table.

a Married includes also persons who classified themselves as having a partner. The unmarried group includes persons who reported that they are divorced, widowed or separated.

A multivariable analysis stratified by population group (Tables 4 and 5) showed that among Jewish participants, those with the least education had the greatest likelihood of infection with both *H. pylori* phenotypes. The associations of sibship size and country of birth with both CagA positive and CagA negative infections persisted (Table 4). Among Arab participants, the associations of education and number of siblings with both CagA positive and negative infections, as well as the association of marital status with CagA negative infections, persisted in a multivariable model (Table 5).

**Table 4.** Associations among Jewish participants, of sociodemographic characteristics with *H. pylori* phenotypes (CagA positive and CagA negative) compared with *H. pylori* sero-negatives^a^

	*H. pylori* positive CagA positive	*H. pylori* positive CagA negative	*H. pylori* positive CagA positive		*H. pylori* positive CagA negative	
	Unadjusted OR (95% CI)	Unadjusted OR (95% CI)	Adjusted OR (95% CI)	*P*	Adjusted OR (95% CI)	*P*
Age (years)	Overall *P* = 0.4, DF = 8		Overall *P* = 0.4, DF = 8			
25–34	0.83 (0.40–1.72)	0.73 (0.41–130)	1.07 (0.48–2.42)	0.8	0.78 (0.41–1.50)	0.4
35–44	0.86 (0.45–1.64)	0.74 (0.44–1.24)	0.99 (0.48–2.03)	0.9	0.71 (0.40–1.27)	0.2
45–54	1.41 (0.76–2.62)	1.15 (0.69–1.91)	1.78 (0.90–3.54)	0.099	1.21 (0.69–2.14)	0.5
55–64	1.23 (0.67–2.26)	1.22 (0.75–1.98)	1.10 (0.57–2.13)	0.7	1.14 (0.67–1.94)	0.6
65–78	1	1	1		1	
Marital status^b^	Overall *P* = 0.4, DF = 2		Overall *P* = 0.6, DF = 2			
Unmarried	1	1	1		1	
Married	1.34 (0.83–2.16)	1.07 (0.74–1.56)	1.27 (0.76–2.12)	0.3	1.00 (0.67–1.51)	0.9
Education	Overall *P* < 0.001, DF = 4		Overall *P* = 0.11, DF = 4			
Some high school or less	2.45 (1.50–4.01)	2.60 (1.75–3.86)	1.84 (1.02–3.30)	0.042	1.68 (1.06–2.67)	0.028
High school certificate/some college	1.58 (0.93–2.69)	1.24 (0.80–1.92)	1.32 (0.74–2.34)	0.3	1.03 (0.64–1.65)	0.9
Academic education	1	1	1		1	
Number of siblings	Overall *P* < 0.001, DF = 4		Overall *P* < 0.001, DF = 4			
0–3	1	1	1		1	
4–7	2.17 (1.33–3.54)	3.10 (2.09–4.60)	1.95 (1.10–3.44)	0.021	2.77 (1.77–4.32)	<0.001
⩾8	3.77 (1.99–7.16)	3.69 (2.10–6.51)	3.20 (1.52–6.72)	0.002	3.19 (1.68–6.04)	<0.001
Country of birth	Overall *P* < 0.001, DF = 12		Overall *P* < 0.001, DF = 12			
Israel	1	1	1		1	
FSU	2.36 (1.25–4.45)	1.34 (0.77–2.39)	4.51 (2.21–9.22)	<0.001	2.61 (1.40–4.84)	0.002
East Europe	1.27 (0.48–3.34)	0.79 (0.34–1.81)	2.13 (0.75–6.03)	0.15	1.14 (0.47–2.78)	0.7
Rest of Europe	0.75 (0.31–1.85)	0.59 (0.29–1.20)	1.17 (0.46–3.02)	0.7	0.93 (0.44–1.96)	0.8
Asia	3.81 (1.80–8.10)	2.07 (1.04–4.14)	3.16 (1.38–7.21)	0.006	1.41 (0.67–2.99)	0.3
Africa	2.52 (1.17–5.41)	2.17 (1.14–4.14)	2.31 (1.00–5.34)	0.05	1.82 (0.89–3.70)	0.099
Americas	0.14 (0.03–0.60)	0.22 (0.10–0.49)	0.19 (0.04–0.82)	0.026	0.29 (1.30–6.80)	0.004

a Multinomial logistic regression model that adjusted for the variables in the table. OR, odds ratio; CI, confidence interval; CagA, cytotoxin-associated gene A; DF, degrees of freedom; FSU, Former Soviet Union.

b Married includes also persons who classified themselves as having a partner. The unmarried group includes persons who reported that they are divorced, widowed or separated.

**Table 5.** Associations among Arab participants, of sociodemographic characteristics with *H. pylori* phenotypes (CagA positive and CagA negative) compared with *H. pylori* sero-negatives^a^

	*H. pylori* positive CagA positive	*H. pylori* positive CagA negative	*H. pylori* positive CagA positive		*H. pylori* positive CagA negative	
	Unadjusted OR (95% CI)	Unadjusted OR (95% CI)	Adjusted OR (95% CI)	*P*	Adjusted OR (95% CI)	*P*
Age (years)	Overall *P* = 0.8, DF = 8		Overall *P* = 0.7, DF = 8			
25–34	1.63 (0.84–3.20)	1.28 (0.67–2.46)	1.58 (0.77–3.23)	0.2	1.10 (0.55–2.18)	0.7
35–44	1.07 (0.60–1.89)	1.06 (0.62–1.82)	0.89 (0.48–1.66)	0.7	0.76 (0.42–1.37)	0.3
45–54	1.15 (0.65–2.01)	1.07 (0.63–1.83)	1.05 (0.57–1.95)	0.8	0.84 (0.47–1.51)	0.5
55–64	1.07 (0.61–1.86)	0.88 (0.52–1.49)	0.97 (0.54–1.74)	0.9	0.73 (0.41–1.27)	0.2
65–78	1	1	1		1	
Marital status^b^	Overall *P* = 0.035, DF = 2		Overall *P* = 0.023, DF = 2			
Unmarried	1	1	1	0.18	1	0.007
Married	1.38 (0.88–2.16)	1.79 (1.15–2.79)	1.38 (0.86–2.22)		1.88 (1.19–2.99)	
Education	Overall *P* = 0.003, DF = 4		Overall *P* = 0.01, DF = 4			
Some high school or less	2.87 (1.67–4.93)	1.75 (1.09–2.81)	2.73 (1.55–4.82)	0.001	1.59 (0.96–2.63)	0.073
High school certificate/some college	2.54 (1.34–4.81)	1.88 (1.06–3.32)	2.39 (1.24–4.59)	0.009	1.67 (0.93–3.00)	0.088
Academic education	1	1	1		1	
Number of siblings	Overall *P* = 0.009, DF = 4		Overall *P* = 0.035, DF = 4			
0–3	1	1	1		1	
4–7	1.56 (0.85–2.84)	1.89 (1.07–3.34)	1.46 (0.78–2.73)	0.2	1.93 (1.07–3.48)	0.03
⩾8	2.56 (1.38–4.72)	2.48 (1.38–4.45)	2.22 (1.15–4.28)	0.017	2.45 (1.31–4.57)	0.005

a Multinomial logistic regression model that adjusted for the variables in the table. OR, odds ratio; CI, confidence intervals CagA, cytotoxin-associated gene A. DF, degrees of freedom.

b Married includes also persons who classified themselves as having a partner. The unmarried group includes persons who reported that they are divorced, widowed or separated.

## Discussion

The main finding of this assessment is the differential prevalence of *H. pylori* phenotype between adult Arabs and Jews living in the same city. The prevalence of *H. pylori* IgG serum antibodies in the Arab sample was higher than in the Jewish sample: 83% *vs.* 61%. The prevalence of infection among Jews aged 25–78 years from Jerusalem was slightly higher than the 56% prevalence found among healthy Israeli blood donors of the same age [5]. The high prevalence of *H. pylori* infection among Arabs is not unexpected. We previously showed 82% *H. pylori* positivity (by means of a stool antigen detection assay) among Arab mothers (median age 33 years) of young children residing in northern Israel [4]. To the best of our knowledge, the large gap in the sero-prevalence of *H. pylori* IgG antibody between healthy adult Arabs and Jews is reported here for the first time. These findings are in line with observations from two sero-surveys conducted among children and adolescents [5, 24]. This difference was only partly explained by disparities in the number of siblings and in educational level, indicating the existence of additional contributing factors.

In our Jewish population sample, the prevalence of *H. pylori* infection varied according to country of birth, with persons born in the FSU, and African and Middle Eastern regions comprising the main risk groups for the infection; whereas the lowest prevalence was found in those from the Americas. This concurs with several studies from Israel [3, 5, 24], Europe [2] and the United States [22] that reported a higher prevalence of *H. pylori* infection in immigrants from endemic countries compared with the native-born population. The Israeli experience of immigration is unique, in the extent of demographic shifts that occurred within a few decades. The Jewish population in Israel increased by 9.3-fold, from 0.64 million in 1948 to 6.1 million in 2013; about 41% of this increase is attributed to the absorption of large waves of immigration [25]. Studies of *H. pylori* infection in developed countries [22, 26] have focused on immigrants from countries with a high prevalence of infection. Our study adds new knowledge regarding immigration from low prevalence to high prevalence settings. Immigrant Jews from Western Europe and North America display a prevalence that more closely resembles that of their families in their countries of origin rather than of the host country. Studies conducted among North American missionaries and U.S. military personnel deployed to developing countries, including the Middle East, have shown quite substantial *H. pylori* IgG antibody sero-conversion of 1.9% to 7.3% per year of exposure [27–29], thus suggesting that adulthood acquisition of the infection is plausible when relocating to endemic regions. Our finding of low prevalence of *H. pylori* infection in persons who were born in the Americas might be explained by low levels of early childhood exposure to infection, and subsequent limited mixing and exposure to *H. pylori* in the new country. Collectively, our results reflect clustering of *H. pylori* infection within families [4, 9], and intrafamilial rather than horizontal transmission of *H. pylori* in a high-income country like Israel [30]. Evidence is conflicting regarding waterborne transmission of *H. pylori* infection [31, 32]. The drinking-water system in Israel is piped. Universal chlorination of drinking water began in the late 1980s. We cannot rule out the possibility that differences in water sources and sanitation systems between Israel/Jerusalem and origin countries of Jewish immigrants might partially explain the association of country of birth with the prevalence of *H. pylori* infection.

Overall, the prevalence of CagA IgG serum antibodies was 38.9% among *H. pylori* sero-positives; 32.5% among Jews and 42.3% among Arabs. This corroborates our previous observation of ~40% among children [24]. Variation across countries in CagA IgG antibody sero-positivity has been documented, with reports ranging from 28% to 82% [22, 33, 34]; this is in addition to within-country variation according to population sub-groups [22, 34]. While such differences might be attributed to variation in detection methods of CagA IgG antibodies, we used a validated in-house ELISA protocol that employed recombinant CagA antigen [10].

We found shared risk factors for infections with CagA positive and negative strains, thus implying common sources and modes of transmission. We found that persons born in the FSU, Western Asia (the Middle East) and Africa had 4.5-, 3.2- and 2.3-fold-increased likelihoods, respectively, of infection with CagA positive strains than persons born in Israel. In parallel, Israeli Jews who were born in the FSU and Asian countries displayed a high risk for non-cardia gastric cancer [35], which is mostly attributable to *H. pylori* infection. In the United States, persons born in developing countries *vs.* industrialised countries, and African-Americans compared with whites, had increased risks of infection with CagA strains [22]. Country of birth may also be a surrogate of unmeasured qualities that can alter the likelihood of exposure to virulent strains or the susceptibility to colonisation with these strains. Further, host-genetic factors may play a role. Indeed, a study based on two cohorts demonstrated an association between genetic loci and *H. pylori* serological status [36]. This prompts an exploration of the impact of host genetic factors on colonisation with specific *H. pylori* phenotypes. Low educational level was a stronger predictor of infections with CagA antigen positive than with non-CagA strains, in both the Arab and Jewish population samples. Current educational level could simply reflect childhood socioeconomic status, a known determinant of *H. pylori* infection acquisition.

Sibship size was shown repeatedly to be positively associated with *H. pylori* infection [2]. In our study, such an association was evident with both CagA positive and negative infections, in both populations; this implies overall enhanced person-to-person transmission of the infection. We found that married individuals compared with unmarried ones, have a higher prevalence of *H. pylori* IgG antibodies; this association was evident for CagA negative strains in the Arab population only. Nonetheless, no significant interaction was found between population group and marital status. Due to the conflicting results regarding the transmission of *H. pylori* infection between couples [37, 38]; further studies are needed to better understand this issue.

*H. pylori* infection is a causative agent of peptic ulcer and gastric cancer. The development of these diseases involves host-related factors and environmental characteristics (reviewed in [11]), as well as infection with CagA positive strains [10]. We identified several groups with increased prevalence of *H. pylori* infection, namely Arabs, less educated persons, those who belong to large families and immigrants from FSU and Asian/African countries. This information is of particular clinical importance and can be used by physicians in the diagnosis and treatment of *H. pylori*-related diseases.

Our study has limitations. As *H. pylori* infection was determined by ELISA measurement of serum IgG antibodies, our findings reflect exposure to *H. pylori* infection, rather than active infection. However, *H. pylori* infection is generally acquired in early life and persists for a lifetime, unless it is treated.

The strengths of this study include the population-based design, in which two distinct populations residing in the same city were sampled; and the same methods and protocols were employed. This enabled valid inference regarding true variations in *H. pylori* infection by phenotype and across populations.

In conclusion, the prevalence of *H. pylori* infection and CagA IgG serum antibodies (which reflect virulence) is higher in the Arab than the Jewish population in Jerusalem. In the Jewish population, the prevalences were highest among persons born in the FSU, Middle East and North Africa and were lowest in those born in Israel and the Americas. This picture likely reflects intrafamilial transmission of the infection, and limited mixing among the various population groups. Future studies are needed to explore the diversity of CagA strains, within each population group and across sub-population group, and the relation to gastroduodenal diseases. Our findings are clinically useful for the detection of risk groups for *H. pylori*-related gastroduodenal illnesses; and highlight the need for particular attention to immigrants from endemic countries and to persons residing in low socioeconomic communities.

## Supplementary material

For supplementary material accompanying this paper visit https://doi.org/10.1017/S0950268819001456. click here to view supplementary material
