# Supplementary material for: Correlates of infection with Helicobacter pylori positive and negative cytotoxin-associated gene A phenotypes among Arab and Jewish residents of Jerusalem
Source: Epidemiol Infect. 2019 Sep 25;147:e276. doi: 10.1017/S0950268819001456 (PMC6807302; doi:10.1017/S0950268819001456)
Supplement: Supplementary file 1 [file S0950268819001456sup001.docx]

**Epidemiology and Infection**

**Determinants of *Helicobacter pylori* infection according to cytotoxin–associated gene A phenotype among Arab and Jewish residents of Jerusalem**

K. MUHSEN ^1^, R. SINNEREICH ^2^, G. BEER-DAVIDSON ^1^, H. NASSAR ^3^, W. ABU AHMED ^2^, D.COHEN ^1 *^, J.D. KARK ^2*^

*These authors contributed equally as senior authors

^1^ Department of Epidemiology and Preventive Medicine, School of Public Health, Sackler Faculty of Medicine, Tel Aviv University Ramat Aviv, Tel Aviv, Israel

^2^ Hebrew University-Hadassah School of Public Health and Community Medicine, Jerusalem, Israel

^3^ St. Joseph Hospital, East Jerusalem and Department of Cardiology, Hadassah-Hebrew University Medical Center, Ein Kerem, Jerusalem 91120, Israel

**Supplementary Material**

**Supplementary table 1: Sero-prevalence of *Helicobacter pylori* immunoglobulin G antibodies among Jewish and Arab adults according to sociodemographic characteristics**

|  | Jews |  | Arabs |  | Total |  |
| --- | --- | --- | --- | --- | --- | --- |
|  | **Positive/total (%)** | **P**^*^ | **Positive/total (%)** | **P**^*^ | **Positive/total (%)** | **P**^*^ |
| Total |  |  |  |  | 1224/1651 (74.1) |  |
| Population |  |  |  |  |  |  |
| Jews | - |  | - |  | 425/692 (61.4) | <0.001 |
| Arabs | - |  | - |  | 799/959 (83.3) |  |
| Sex |  |  |  |  |  |  |
| Men | 229/365 (62.7) | 0.4 | 420/509 (82.5) | 0.4 | 649/874 (74.3) | 0.9 |
| Women | 196/327 (59.9) |  | 379/450 (84.2) |  | 575/777 (74.0) |  |
| Age (years)^†^ |  | 0.15 |  | 0.7 |  | 0.7 |
| 25-34 | 48/88 (54.5) |  | 113/130 (86.9)) |  | 161/218 (73.9) |  |
| 35-44 | 71/129 (55.0) |  | 164/197 (83.2) |  | 235/326 (72.1) |  |
| 45-54 | 97/147 (66.0) |  | 175/209 (83.7) |  | 272/356 (76.4) |  |
| 55-64 | 114/173 (65.9) |  | 165/202 (81.7) |  | 279/375 (74.4) |  |
| 65-78 | 95/155 (61.3) |  | 182/221 (82.4) |  | 277/ 376 (73.7) |  |
| Marital status^**^ |  |  |  |  |  |  |
| Married | 317/508 (62.4) | 0.4 | 662/783 (84.5) | 0.024 | 979/1291 (75.8) | 0.003 |
| Unmarried | 107/181 (59.1) |  | 134/173 (77.5) |  | 241/354 (68.1) |  |
| Education^§^ |  | <0.001 |  | 0.004 |  | <0.001 |
| Some high school or less | 193/264 (73.1) |  | 523/615 (85.0) |  | 716/879 (81.5) |  |
| High school certificate/some college | 99/169 (58.9) |  | 180/212 (84.9) |  | 279/380 (73.4) |  |
| Academic education | 132/256 (51.6) |  | 94/129 (72.9) |  | 226/385 (58.7) |  |
| Religiosity |  | 0.071 |  | 0.7 |  | 0.2 |
| Religious/ very religious | 150/262 (57.3) |  | 302/360 (83.9) |  | 452/622 (72.7) |  |
| Traditional/secular | 272/424 (64.2) |  | 493/594 (83.0) |  | 765/1018 (75.1) |  |
| Number of siblings‡ |  |  |  |  |  |  |
| 0-3 | 194/384 (50.5) | <0.001 | 66/91 (72.5) | 0.002 | 260/475 (54.7) | <0.001 |
| 4-7 | 153/207 (73.9) |  | 354/431 (82.1) |  | 507/ 638 (79.5) |  |
| ≥8 | 76/96 (79.2) |  | 378/435 (86.9) |  | 454/531 (85.5) |  |
| Country of birth^†^ |  |  |  |  |  |  |
| Israel | 216/360 (60.0) | <0.001 | - |  | - |  |
| Former Soviet Union | 59/83 (71.1) |  | - |  | - |  |
| East Europe | 18/31 (58.1) |  |  |  |  |  |
| Rest of Europe | 21/43 (48.8) |  |  |  |  |  |
| Asia | 50/63 (79.4) |  | - |  | - |  |
| Africa | 51/66 (77.3) |  | - |  | - |  |
| Americas | 10/44 (22.7) |  | - |  | - |  |

^*^ P values was obtained by chi square test, unless specified otherwise ^§^ P for trend

^†^ Degrees of freedom (DF) =4 ‡ DF =2

^**^ Married includes also persons who classified themselves as having a partner. The unmarried group includes participants who reported that they are divorced, widowed or separated.
